# (*E*)-2-(2-Furylmethyl­idene)-2,3-dihydro-1*H*-pyrrolizin-1-one

**DOI:** 10.1107/S1600536810017939

**Published:** 2010-06-05

**Authors:** Yousaf Ali, Peng Yu, Erbing Hua, Guo Rui, Sun Qi

**Affiliations:** a Department of Pharmaceutical Engineering, Biotechnology College, Tianjin University of Science & Technology (TUST), Tianjin 300457, People’s Republic of China

## Abstract

The title compound, C_12_H_9_NO_2_, was prepared by an Aldol reaction of furfuraldehyde with 2,3-dihydro-1*H*-pyrrolizin-1-one. The mol­ecule is almost planar, with an r.m.s. deviation of 0.045 Å, excluding the methyl­ene H atoms. In the crystal structure, mol­ecules are linked *via* weak inter­molecular C—H⋯O hydrogen bonding and aromatic π–π stacking [centroid–centroid distance = 3.6151 (9) Å].

## Related literature

For general background to synthetic dihydro­pyrrolizines and for the biological activity of related structures, see: Meinwald & Meinwald (1965 ▶); Skvortsov & Astakhova (1992 ▶). For the preparation of the starting material, see: Clemo & Ramage (1931 ▶); Braunholtz *et al.* (1962 ▶).
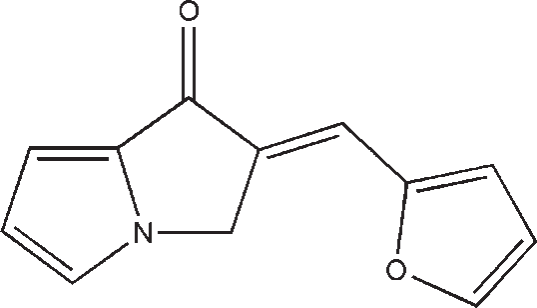


## Experimental

### 

#### Crystal data

C_12_H_9_NO_2_
*M* *_r_* = 199.20
Monoclinic, 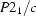

*a* = 11.8170 (16) Å
*b* = 6.1242 (6) Å
*c* = 14.432 (2) Å
β = 113.157 (3)°
*V* = 960.3 (2) Å^3^
*Z* = 4
Mo *K*α radiation
μ = 0.10 mm^−1^
*T* = 113 K
0.22 × 0.18 × 0.12 mm

#### Data collection

Rigaku Saturn724 CCD camera diffractometer
9348 measured reflections
2271 independent reflections
1796 reflections with *I* > 2σ(*I*)
*R*_int_ = 0.025

#### Refinement

*R*[*F* ^2^ > 2σ(*F* ^2^)] = 0.036
*wR*(*F* ^2^) = 0.100
*S* = 1.07
2271 reflections
136 parameters
H-atom parameters constrained
Δρ_max_ = 0.24 e Å^−3^
Δρ_min_ = −0.23 e Å^−3^

### 

Data collection: *CrystalClear* (Rigaku, 2009 ▶); cell refinement: *CrystalClear*; data reduction: *CrystalClear*; program(s) used to solve structure: *SHELXS97* (Sheldrick, 2008 ▶); program(s) used to refine structure: *SHELXL97* (Sheldrick, 2008 ▶); molecular graphics: *ORTEP-3* (Farrugia, 1997 ▶); software used to prepare material for publication: *publCIF* (Westrip, 2010 ▶).

## Supplementary Material

Crystal structure: contains datablocks I, global. DOI: 10.1107/S1600536810017939/xu2760sup1.cif

Structure factors: contains datablocks I. DOI: 10.1107/S1600536810017939/xu2760Isup2.hkl

Additional supplementary materials:  crystallographic information; 3D view; checkCIF report

## Figures and Tables

**Table 1:** Hydrogen-bond geometry (Å, °)

*D*—H⋯*A*	*D*—H	H⋯*A*	*D*⋯*A*	*D*—H⋯*A*
C6—H6*A*⋯O1^i^	0.99	2.47	3.3076 (15)	142
C8—H8⋯O1^ii^	0.95	2.55	3.3096 (14)	137
C10—H10⋯O1^ii^	0.95	2.46	3.1789 (15)	133

Symmetry codes: (i) 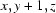; (ii) 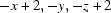.

**Fig. 1.**
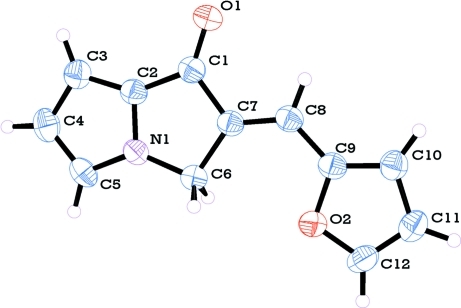
View of the single molecule showing atom numbering scheme. Displacement ellipsoids are drawn at 80% probability level.

**Fig. 2.**
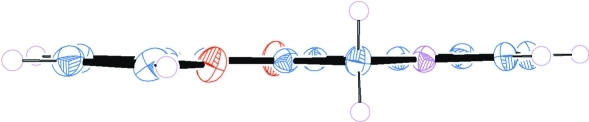
View of molecule from top, showing two H atoms of methylene group, one above and one below the rings.

**Fig. 3.**
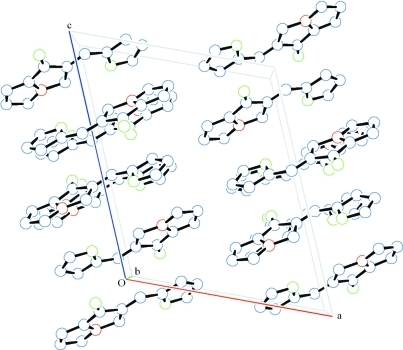
Packing diagram of cell unit showing straight chains not parallel to each other.

**Fig. 4.**
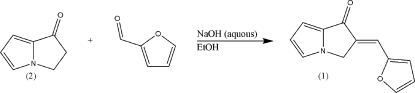
The preparation of the title compound.

## supplementary crystallographic information

### Comment

Derivatives of 2,3-dihydropyrrolizine became known through studies of their synthesis (Clemo & Ramage, 1931; Braunholtz *et al.*, 1962) and isolation from natural source (Meinwald & Meinwald, 1965). Synthetic dihydropyrrolizines that are of interest as pharmaceuticals have been reported. The most important of these, Ketorolac, is a non steroid analgesic. Depending on their structure, derivatives of 2,3-dihydropyrrolizine have shown merit as analgesics, anti-inflammatory agents, myorelaxants, inhibitors of thrombocyte aggregation, fibrinolytics, temperature-lowering substances and drugs for the treatment of glaucoma and conjunctivitis (Skvortsov & Astakhova, 1992).

Numbering scheme for the title compound is shown in an ORTEP (Farrugia, 1997) plot of the molecule, see Fig. 1. The three rings are essentially planar, rms deviation = 0.045 Å, with two metnylene H atoms above and below the plane (Fig. 2). Weak intermolecular C–H···O hydrogen bonding (Table 1) and aromatic π-π stacking between O2-containg ring and N1-containg ring [the centroids distance 3.6151 (9) Å] are present in the crystal structure (Fig. 3). Double bond connecting two ring systems have an E configuration.

### Experimental

Title compound was prepared by an Aldol reaction of furfuraldehyde with 2,3-dihydro-1*H*-pyrrolizin-1-one (Fig. 4). Purification was carried out by Flash Column Chromatography, Petroleum Ether : Ethyl Acetate = 3:1, followed by recrystalization from Petroleum Ether.

### Refinement

All H atoms were placed in geometrically idealized positions and constrained to ride on their parent atoms with C—H = 0.95 and 0.99 Å for aromatic and methylene respectively. U_iso_ (H) values were taken to be equal to 1.2 U_eq_(C) for all hydrogen atoms.

### Crystal data

**Table:**

C_12_H_9_NO_2_	*F*(000) = 416
*M**_r_* = 199.20	*D*_x_ = 1.378 Mg m^−^^3^
Monoclinic, *P*2_1_/*c*	Mo *K*α radiation, λ = 0.71075 Å
Hall symbol: -P 2ybc	Cell parameters from 2971 reflections
*a* = 11.8170 (16) Å	θ = 1.5–28.0°
*b* = 6.1242 (6) Å	µ = 0.10 mm^−^^1^
*c* = 14.432 (2) Å	*T* = 113 K
β = 113.157 (3)°	Prism, colourless
*V* = 960.3 (2) Å^3^	0.22 × 0.18 × 0.12 mm
*Z* = 4	

### Data collection

**Table:**

Rigaku Saturn724 CCD camera diffractometer	1796 reflections with *I* > 2σ(*I*)
Radiation source: rotating anode	*R*_int_ = 0.025
multilayer	θ_max_ = 27.9°, θ_min_ = 1.9°
ω scans	*h* = −14→15
9348 measured reflections	*k* = −7→7
2271 independent reflections	*l* = −18→18

### Refinement

**Table:**

Refinement on *F*^2^	Primary atom site location: structure-invariant direct methods
Least-squares matrix: full	Secondary atom site location: difference Fourier map
*R*[*F*^2^ > 2σ(*F*^2^)] = 0.036	Hydrogen site location: inferred from neighbouring sites
*wR*(*F*^2^) = 0.100	H-atom parameters constrained
*S* = 1.07	*w* = 1/[σ^2^(*F*_o_^2^) + (0.0572*P*)^2^ + 0.0917*P*] where *P* = (*F*_o_^2^ + 2*F*_c_^2^)/3
2271 reflections	(Δ/σ)_max_ = 0.001
136 parameters	Δρ_max_ = 0.24 e Å^−^^3^
0 restraints	Δρ_min_ = −0.23 e Å^−^^3^

### Special details

**Table:**

Experimental. Single crystals suitable for X-ray crystallography were grown by slow evaporatin from ethyl acetate solution and of by slow cooling of a hot saturated solution of Petroleum Ether. Crystals obtained from later were found more suitable for X ray analysis.
Geometry. All esds (except the esd in the dihedral angle between two l.s. planes) are estimated using the full covariance matrix. The cell esds are taken into account individually in the estimation of esds in distances, angles and torsion angles; correlations between esds in cell parameters are only used when they are defined by crystal symmetry. An approximate (isotropic) treatment of cell esds is used for estimating esds involving l.s. planes.
Refinement. Refinement of *F*^2^ against ALL reflections. The weighted *R*-factor wR and goodness of fit *S* are based on *F*^2^, conventional *R*-factors *R* are based on *F*, with *F* set to zero for negative *F*^2^. The threshold expression of *F*^2^ > σ(*F*^2^) is used only for calculating *R*-factors(gt) etc. and is not relevant to the choice of reflections for refinement. *R*-factors based on *F*^2^ are statistically about twice as large as those based on *F*, and *R*- factors based on ALL data will be even larger.

### Fractional atomic coordinates and isotropic or equivalent isotropic displacement parameters (Å^2^)

**Table:**

	*x*	*y*	*z*	*U*_iso_*/*U*_eq_	
O1	0.81898 (7)	0.03138 (13)	0.87581 (6)	0.0268 (2)	
O2	1.15722 (7)	0.60788 (13)	0.91369 (6)	0.0239 (2)	
N1	0.76145 (8)	0.52826 (15)	0.74753 (7)	0.0190 (2)	
C1	0.82061 (10)	0.21177 (18)	0.83928 (8)	0.0190 (2)	
C2	0.71886 (10)	0.33225 (17)	0.76750 (8)	0.0187 (2)	
C3	0.59277 (10)	0.32408 (19)	0.71216 (8)	0.0219 (3)	
H3	0.5387	0.2083	0.7110	0.026*	
C4	0.56100 (11)	0.52063 (19)	0.65837 (9)	0.0254 (3)	
H4	0.4806	0.5618	0.6133	0.030*	
C5	0.66732 (10)	0.64550 (18)	0.68219 (8)	0.0229 (3)	
H5	0.6725	0.7869	0.6571	0.027*	
C6	0.89436 (10)	0.56200 (19)	0.80194 (8)	0.0209 (2)	
H6A	0.9119	0.6913	0.8466	0.025*	
H6B	0.9363	0.5784	0.7550	0.025*	
C7	0.93162 (10)	0.35275 (17)	0.86190 (8)	0.0182 (2)	
C8	1.04282 (10)	0.29109 (18)	0.92790 (8)	0.0194 (2)	
H8	1.0468	0.1525	0.9586	0.023*	
C9	1.15627 (10)	0.40818 (18)	0.95782 (8)	0.0197 (2)	
C10	1.27159 (10)	0.35966 (18)	1.02555 (8)	0.0221 (3)	
H10	1.2959	0.2323	1.0662	0.026*	
C11	1.34842 (10)	0.5362 (2)	1.02364 (8)	0.0238 (3)	
H11	1.4342	0.5499	1.0626	0.029*	
C12	1.27594 (10)	0.6803 (2)	0.95590 (8)	0.0247 (3)	
H12	1.3037	0.8148	0.9396	0.030*	

### Atomic displacement parameters (Å^2^)

**Table:**

	*U*^11^	*U*^22^	*U*^33^	*U*^12^	*U*^13^	*U*^23^
O1	0.0259 (4)	0.0196 (4)	0.0298 (4)	−0.0021 (3)	0.0056 (4)	0.0068 (3)
O2	0.0229 (4)	0.0244 (4)	0.0219 (4)	−0.0043 (3)	0.0061 (3)	0.0043 (3)
N1	0.0198 (5)	0.0185 (5)	0.0183 (4)	0.0014 (4)	0.0070 (4)	0.0023 (4)
C1	0.0218 (6)	0.0175 (5)	0.0178 (5)	−0.0001 (4)	0.0079 (4)	−0.0011 (4)
C2	0.0212 (6)	0.0179 (5)	0.0180 (5)	0.0000 (4)	0.0087 (4)	0.0000 (4)
C3	0.0206 (6)	0.0244 (6)	0.0208 (5)	0.0000 (4)	0.0082 (4)	−0.0007 (4)
C4	0.0216 (6)	0.0292 (7)	0.0236 (5)	0.0067 (5)	0.0070 (5)	0.0015 (5)
C5	0.0256 (6)	0.0204 (6)	0.0218 (5)	0.0066 (5)	0.0084 (5)	0.0044 (4)
C6	0.0202 (6)	0.0203 (6)	0.0212 (5)	−0.0011 (4)	0.0072 (4)	0.0029 (4)
C7	0.0203 (6)	0.0177 (5)	0.0174 (5)	−0.0001 (4)	0.0083 (4)	0.0002 (4)
C8	0.0219 (5)	0.0187 (5)	0.0182 (5)	0.0002 (4)	0.0085 (4)	0.0006 (4)
C9	0.0227 (6)	0.0192 (5)	0.0186 (5)	−0.0001 (4)	0.0095 (4)	0.0002 (4)
C10	0.0211 (6)	0.0237 (6)	0.0207 (5)	−0.0003 (4)	0.0075 (4)	−0.0009 (4)
C11	0.0198 (6)	0.0297 (6)	0.0217 (5)	−0.0037 (5)	0.0078 (4)	−0.0036 (5)
C12	0.0236 (6)	0.0286 (6)	0.0222 (5)	−0.0090 (5)	0.0094 (5)	−0.0020 (5)

### Geometric parameters (Å, °)

**Table:**

O1—C1	1.2274 (13)	C5—H5	0.9500
O2—C12	1.3652 (14)	C6—C7	1.5117 (14)
O2—C9	1.3810 (13)	C6—H6A	0.9900
N1—C5	1.3480 (14)	C6—H6B	0.9900
N1—C2	1.3754 (14)	C7—C8	1.3388 (14)
N1—C6	1.4683 (14)	C8—C9	1.4292 (15)
C1—C2	1.4435 (14)	C8—H8	0.9500
C1—C7	1.4953 (15)	C9—C10	1.3613 (15)
C2—C3	1.3873 (15)	C10—C11	1.4191 (16)
C3—C4	1.4011 (15)	C10—H10	0.9500
C3—H3	0.9500	C11—C12	1.3451 (17)
C4—C5	1.3935 (17)	C11—H11	0.9500
C4—H4	0.9500	C12—H12	0.9500
			
C12—O2—C9	105.97 (9)	N1—C6—H6B	111.5
C5—N1—C2	110.01 (9)	C7—C6—H6B	111.5
C5—N1—C6	135.57 (10)	H6A—C6—H6B	109.3
C2—N1—C6	114.42 (9)	C8—C7—C1	121.59 (10)
O1—C1—C2	128.24 (10)	C8—C7—C6	129.04 (10)
O1—C1—C7	125.91 (10)	C1—C7—C6	109.36 (9)
C2—C1—C7	105.85 (9)	C7—C8—C9	127.96 (11)
N1—C2—C3	108.03 (9)	C7—C8—H8	116.0
N1—C2—C1	109.05 (9)	C9—C8—H8	116.0
C3—C2—C1	142.91 (10)	C10—C9—O2	109.70 (10)
C2—C3—C4	106.33 (10)	C10—C9—C8	131.50 (11)
C2—C3—H3	126.8	O2—C9—C8	118.80 (9)
C4—C3—H3	126.8	C9—C10—C11	106.69 (10)
C5—C4—C3	108.40 (10)	C9—C10—H10	126.7
C5—C4—H4	125.8	C11—C10—H10	126.7
C3—C4—H4	125.8	C12—C11—C10	106.53 (10)
N1—C5—C4	107.23 (10)	C12—C11—H11	126.7
N1—C5—H5	126.4	C10—C11—H11	126.7
C4—C5—H5	126.4	C11—C12—O2	111.11 (10)
N1—C6—C7	101.32 (8)	C11—C12—H12	124.4
N1—C6—H6A	111.5	O2—C12—H12	124.4
C7—C6—H6A	111.5		
			
C5—N1—C2—C3	0.38 (12)	C2—C1—C7—C8	178.79 (10)
C6—N1—C2—C3	179.39 (9)	O1—C1—C7—C6	−179.66 (11)
C5—N1—C2—C1	−178.70 (9)	C2—C1—C7—C6	0.05 (12)
C6—N1—C2—C1	0.31 (12)	N1—C6—C7—C8	−178.51 (11)
O1—C1—C2—N1	179.49 (11)	N1—C6—C7—C1	0.11 (11)
C7—C1—C2—N1	−0.21 (12)	C1—C7—C8—C9	−179.33 (10)
O1—C1—C2—C3	0.9 (2)	C6—C7—C8—C9	−0.85 (19)
C7—C1—C2—C3	−178.76 (14)	C12—O2—C9—C10	0.11 (12)
N1—C2—C3—C4	0.06 (12)	C12—O2—C9—C8	−179.84 (10)
C1—C2—C3—C4	178.62 (14)	C7—C8—C9—C10	178.22 (11)
C2—C3—C4—C5	−0.46 (12)	C7—C8—C9—O2	−1.85 (17)
C2—N1—C5—C4	−0.67 (13)	O2—C9—C10—C11	−0.21 (12)
C6—N1—C5—C4	−179.37 (11)	C8—C9—C10—C11	179.73 (11)
C3—C4—C5—N1	0.69 (13)	C9—C10—C11—C12	0.23 (13)
C5—N1—C6—C7	178.40 (12)	C10—C11—C12—O2	−0.17 (13)
C2—N1—C6—C7	−0.26 (11)	C9—O2—C12—C11	0.04 (12)
O1—C1—C7—C8	−0.91 (17)		

### Hydrogen-bond geometry (Å, °)

**Table:**

*D*—H···*A*	*D*—H	H···*A*	*D*···*A*	*D*—H···*A*
C6—H6A···O1^i^	0.99	2.47	3.3076 (15)	142
C8—H8···O1^ii^	0.95	2.55	3.3096 (14)	137
C10—H10···O1^ii^	0.95	2.46	3.1789 (15)	133

Symmetry codes: (i) *x*, *y*+1, *z*; (ii) −*x*+2, −*y*, −*z*+2.
